# Properties and Mechanisms of Flavin-Dependent Monooxygenases and Their Applications in Natural Product Synthesis

**DOI:** 10.3390/ijms23052622

**Published:** 2022-02-27

**Authors:** Yaming Deng, Quan Zhou, Yuzhou Wu, Xi Chen, Fangrui Zhong

**Affiliations:** 1 Key Laboratory of Synthetic and Natural Functional Molecule of the Ministry of Education, College of Chemistry and Materials Science, Northwest University, Xi’an 710127, China; 2021612300@hust.edu.cn (Y.D.); 201820711@stumail.nwu.edu.cn (Q.Z.); 2 Key Laboratory of Material Chemistry for Energy Conversion and Storage, Ministry of Education, Hubei Engineering Research Center for Biomaterials and Medical Protective Materials, Hubei Key Laboratory of Bioinorganic Chemistry & Materia Medica, School of Chemistry and Chemical Engineering, Huazhong University of Science and Technology (HUST), 1037 Luoyu Road, Wuhan 430074, China; wuyuzhou@hust.edu.cn

**Keywords:** natural products, flavin monooxygenases (FMOs), flavin-C4a-(hydrogen) peroxide, flavin-N5-oxide

## Abstract

Natural products are usually highly complicated organic molecules with special scaffolds, and they are an important resource in medicine. Natural products with complicated structures are produced by enzymes, and this is still a challenging research field, its mechanisms requiring detailed methods for elucidation. Flavin adenine dinucleotide (FAD)-dependent monooxygenases (FMOs) catalyze many oxidation reactions with chemo-, regio-, and stereo-selectivity, and they are involved in the synthesis of many natural products. In this review, we introduce the mechanisms for different FMOs, with the classical FAD (C4a)-hydroperoxide as the major oxidant. We also summarize the difference between FMOs and cytochrome P450 (CYP450) monooxygenases emphasizing the advantages of FMOs and their specificity for substrates. Finally, we present examples of FMO-catalyzed synthesis of natural products. Based on these explanations, this review will expand our knowledge of FMOs as powerful enzymes, as well as implementation of the FMOs as effective tools for biosynthesis.

## 1. Introduction

Today, natural products (NPs) play increasingly important roles, especially in early drug development [1,2]. The synthetic pathways of these natural products include a variety of enzymes with different functions. However, the chemical synthesis required to achieve this goal is difficult [3]. To break this bottleneck, scientists have studied in depth the natural product synthesis pathways for terpenoids, non-ribosomal peptides, and polyketides [4]. They have discovered that most secondary metabolites’ synthesis pathways depend on the core enzymes such as polyketide synthase (PKS) and non-ribosomal peptide synthase (NRP) [4]. In addition, extra tailoring enzymes are required to refine the structure of natural products in order to modify the substrates precisely and promote their bioactivities, such as glycosylation (C, O, or N), halogenation, acylation, alkylation, or redox reactions (de-hydroxylation, saturation, or epoxidation) [5,6]. A large number of combinatorial enzymes can carry out precise regional and stereoselective modification on complex substrates, which are usually difficult to achieve by chemical methods [7,8]. Therefore, combinatorial biology is a tool that can carry out the most diverse and complex structural modifications of natural products [9]. However, compared to nature’s wisdom, there is still a long way to go for scientists to understand and apply natural enzymes for synthesis [10]. In nature, there are many enzymes utilizing oxygen as a substrate and metal as a cofactor for synthesis. One class of these enzymes depends on flavin but not metal. Flavin is a widely used redox cofactor in the form of flavin mononucleotide (FMN) or flavin adenine dinucleotide (FAD). There are three classes of these enzymes: flavin dependent dehydrogenase/reductase, oxidase, and monooxygenase (FMO). Compared with flavin dependent oxidase, which uses O_2_ as electron acceptor, flavin monooxygenase usually uses nicotinamide adenine dinucleotide phosphate (NAD(P)H) as a hydrogen donor to catalyze the binding of oxygen atoms in molecular oxygen to small molecules by forming C4a-(hydrogen) peroxide, as an intermediate stage in producing natural products with chemo-, regio- and enantio-selectivity. Therefore, FMO is an important oxidoreductase. Their application as biocatalysts in biotechnology and the pharmaceutical industry will greatly promote the development of drugs [11].

There have been some excellent reviews in the field. In 2017, Tang et al. systematically reviewed different types of oxidative cyclization reactions in natural product biosynthesis, involving non-free radical cyclization catalyzed by FMOs and NAD(P)H-dependent reductases [12]. In the same year, Teufel et al. discussed oxygenases with FAD and FMN as cofactors, which synthesize complex natural products through the catalytic oxidation-reduction of substrates [13]. In 2018, Mattevi et al. reviewed the molecular basis of FMOs in the activation of O_2_. This paper mainly analyzed how O_2_ reaches the flavin cofactor in the protein pocket through a special pathway, as well as the detailed mechanism of FMO catalyzed substrate oxidation [14]. In 2021, Paul et al. made a very comprehensive review of groups A to H FMOs with many concrete examples [15]. In contrast to these, our review focuses on the application of FMOs in natural product synthesis, their differences to CYP450 monooxygenases, and their advantages in natural product synthesis.

## 2. The Characteristics and Functional Mechanisms of FMOs

**Scheme 1:**
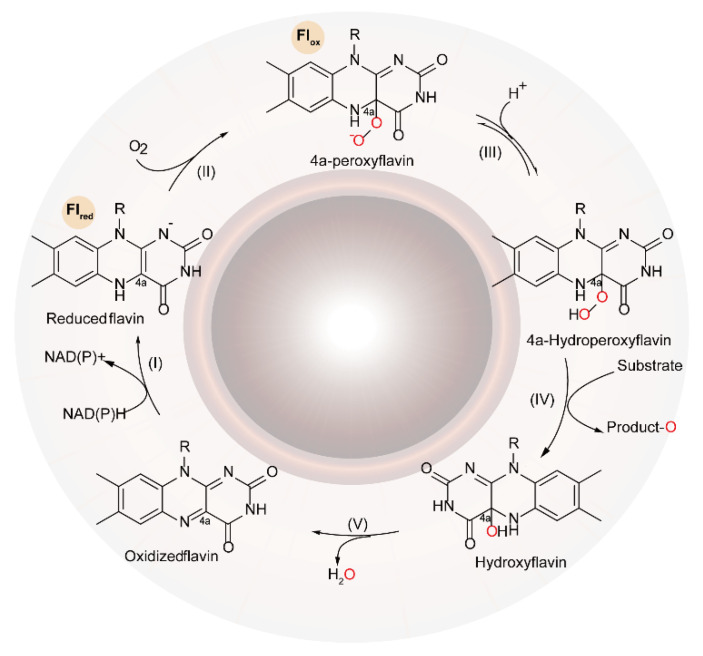
Simplified catalytic cycle of flavin-dependent monooxygenase.

In the early years, scientists thought FMOs could protect animals, preventing damage by foreign organisms [13]. In the 1970s, Ziegler and Poulsen discovered two mammalian FMO enzymes in pig liver microsomes. They described the characteristics and functions of these FMOs. Therefore, FMO was called Ziegler’s enzyme for a period of time. FMOs are flavin proteins containing a single FAD as a cofactor [13]. Microsomal NAD(P)H and molecular oxygen are required in the process of catalytic oxidative metabolism. Flavin is mainly used as an electronic medium to bind to the enzyme, where it usually circulates between the oxidation state and reduction state for electron transfer. FMOs consume molecular oxygen to catalyze the insertion of one oxygen atom to an organic substrate, and the other oxygen atom is reduced to water (Scheme 1). The substrates for FMO catalysis include a variety of compounds containing nucleophilic heteroatoms of nitrogen, sulfur, phosphorus, and selenium [16]. At the same time, FMOs also have a great value in industry [11,17].

### 2.1. The Catalytic Mechanism for One-Component FMOs

**Scheme 2:**
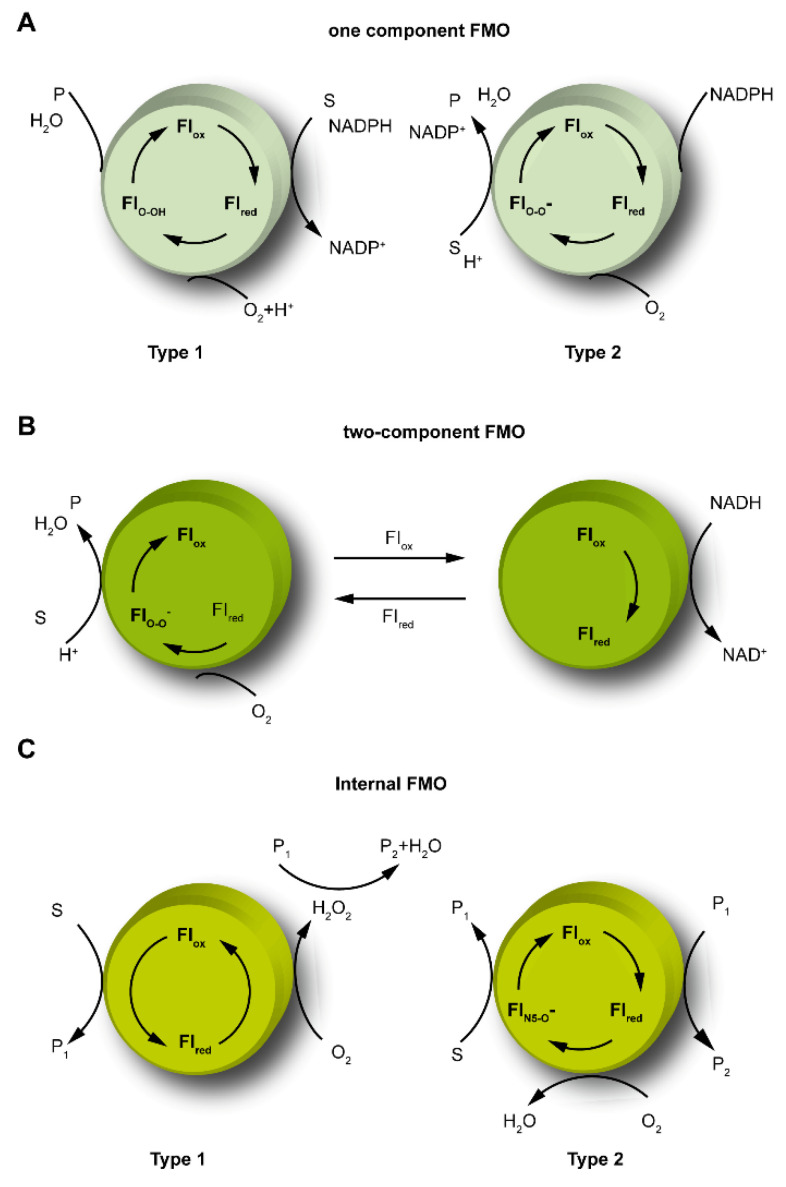
Flavin-dependent monooxygenase (FMOs) catalytic mechanism. There are three classes: (**A**) One-component monooxygenases, in which each half reaction occurs at the same active sites. (**B**) Two-component monooxygenases, in which each half reaction occurs at different active sites. (**C**) Internal monooxygenases, with flavin-N5-oxygen adduct (fla-vin-N5-oxide) as an oxygen-containing substance. S is substrate, P is product, FI is flavin (Fl_ox_: oxidized flavin, Fl_red_: reduced flavin), and Fl_N5-o_^−^ is flavin-N5-oxide.

The catalytic cycle of FMOs is shown in Scheme 1 and Scheme 2A. In the first step of the FMO catalytic cycle, the hydrogen donor NAD(P)H cofactor is bound to the enzyme, and then two electrons are transferred to FAD to reduce it, which is the reduction half reaction to reduce the xanthin cofactor. In step 2, molecular oxygen reacts rapidly with reduced FAD to form xanthin hydroperoxide, which then oxidizes the substrate, and the O–O bond breaks. One oxygen atom inserts into the substrate, and the oxidized substrate then leaves the cycle, while the other oxygen atom forms water; then, NAD(P)^+^ is released from the enzyme to leave the cycle, and, at the same time, the reduced flavin circulates back to the static oxidation state. The most important feature of the catalytic cycle of one-component FMOs is that flavin cofactors usually remain at the same active sites of the enzyme throughout the catalytic cycle, these active sites are composed of dinucleotide binding domains specifically for FAD binding, and some FMO active sites also contain dinucleotide binding domains for NAD(P)H [14,16,18]. Thus, this can avoid the non-specific reaction between reduced flavin and oxygen. However, the disadvantage of one-component FMOs is that this nonspecific reaction will lead to a waste of energy consumption, because it will continue to use physiological energy, such as NADH or NADPH [18].

### 2.2. The Catalytic Mechanism for Two-Component FMOs

The catalytic cycle of two-component FMOs is similar to that of one-component FMOs. The difference is that, in the catalytic cycle of two-component FMOs, each half reaction occurs at different active sites (Scheme 2B) [14]. These enzymes are rich in nature, generally involved in the catalysis of natural product synthesis, metabolism of aliphatic and aromatic compounds, antibiotic synthesis, etc. [19]. Two-component FMOs require two isolated proteins for the whole catalytic cycle, including an NAD(P)H dependent reductase and a catalytic oxidation monooxygenase, in which the reductase reduces the flavin by transferring two electrons by NAD(P)H and then transfers the reduced flavin to the monooxygenase component [20,21]. In the monooxygenase component, the monooxygenase oxidizes the substrate by consuming oxygen. The most common example of two-component FMO is bacterial luciferase, in which the oxygenase component catalyzes the oxidation of long-chain fatty aldehydes to produce luminescent carboxylic acids. Most two-component flavin dependent monooxygenase systems do not need an external addition of flavin in the process of catalysis. Because the flavin cofactor in the reductase component is reduced by NAD(P)H and then transferred to monooxygenase, flavin can be recycled repeatedly in this process [22,23].

However, it is known that organisms containing FMO generally live in an oxygen rich environment, and the reduced flavin is very easily oxidized by the oxygen in the process of transmission to produce H_2_O_2_ and other reactive oxygen species, which are often unfavorable to organisms [18]. Therefore, the production and transfer of reducing flavin on reductase to oxygenase needs to avoid or minimize automatic oxidation. As a result, the diffusion rate of reducing flavin and the binding rate of oxygenase components must be greater than the reaction rate of free reducing flavin and molecular oxygen, so as to minimize the waste of reducing flavin and the formation of hydrogen peroxide and other reactive oxygen species [14]. After being reduced by reductase, flavin can reach oxygenase in two ways, protein–protein complex formation or free diffusion. It was found that the reduced flavin transfer in two-component FMOs mainly followed the free diffusion model. However, some reduced flavin transfer is carried out through protein–protein complexes [18]. Similar to the one-component FMOs, the reactions catalyzed by two-component FMOs usually display significant chemo-, regio- and/or stereo-specificity. Therefore, these enzymes are very promising in biocatalytic application in the synthesis of valuable compounds [22,24].

### 2.3. The Catalytic Mechanism for Internal FMOs

**Scheme 3:**
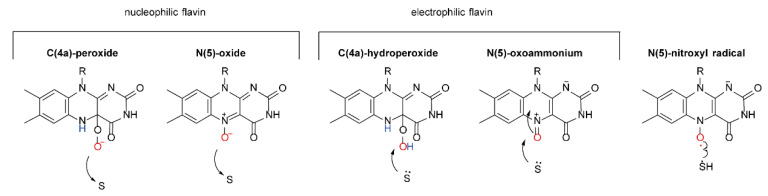
Proposed flavin−oxygen adducts reacting with a substrate in monooxygenases. R is ribityl adenosine diphosphate for FAD and phosphor-ibityl for FMN. S is substrate.

**Scheme 4:**
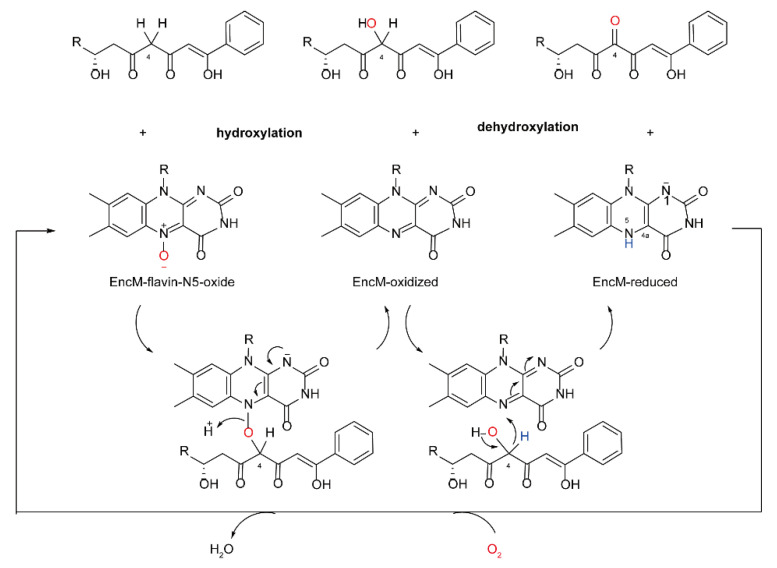
Catalytic Mechanism of Encm with a Polyketide substrate.

The mechanism of internal FMOs was discovered and proposed by Teufel et al. in the study of enterocin biosynthetic enzyme (Encm). They found that the flavin-dependent protein Encm from bacteria can catalyze both the oxidation and dehydrogenation of the substrate, which is different from the classical formation of the C4a-(hydro) peroxy-flavin intermediate [25,26]. The intermediate involved in the double oxidation process of substrate catalyzed by Encm is flavin-N5-oxygen adduct (flavin-N5-oxide) as an oxygen-containing substance (Scheme 3). The intermediate was identified by mass spectrometry, spectrum, and isotope labeling, and it was determined that flavin-N5-oxide was generated in the process. It was found that the flavin-N5-oxide intermediate in Encm was very stable and could stay for at least 7 days at 4 °C. The mechanism of flavin-N5-oxide in Encm was proposed as follows (Scheme 2C, Scheme 4).

First, FAD reduced in Encm transfers flavin N5 to an oxygen molecule to produce protonated superoxide and anionic flavin semiquinone. Then, flavin is converted to flavin-NC5-peroxide, which forms N5 hydroxylamine through water loss. Next, the N5 hydroxylamine produces electrophilic ammonium oxide followed by tautomerization. When interacting with the substrate, the electrophilic ammonium oxide will transfer the oxygen in ammonium oxide to the enolated substrate to oxidize the substrate. At the same time, the oxidized substrate now acts as an electron donor and transfers a proton to the xanthin cofactor to afford the reduced xanthin cofactor. The reduced xanthin cofactor then reacts with O_2_ to obtain flavin N5 oxide, so as to prepare for the next catalytic cycle [25]. The hydroxylation and dehydrogenation catalyzed by Encm are carried out without an external reductant, so Encm can be regarded as an internal monooxygenase [14].

## 3. The Similarities and Differences between FMOs and CYP450

CYP450 enzymes are heme *b*-containing monooxygenases [27]. Heme is a prosthetic group consisting of iron ions coordinated by four porphyrin nitrogen atoms. CYP450 can catalyze various types of reactions, such as epoxidation of C=C double bonds, aromatic hydroxylation, deamination and dehalogenation [28], Baeyer-Villiger oxidation [29], and many other types of reactions with a prosthetic heme and one iron ion in different redox states. With very few exceptions, the catalytic process of CYP450 is as follows; CYP450 first catalyzes the cleavage of the O–O bond of molecular oxygen, thereby introducing one oxygen atom into the substrate, and the other oxygen atom is reduced to water. At the same time, redox partner proteins must be involved in the catalysis of CYP450, which play catalytic functions by transferring electrons from NAD(P)H to the heme center of CYP450 [30].

Both FMOs and CYP450 play an important role in natural product biosynthesis. They share great similarities in catalyzing the oxidation of natural products [31]. First, similar to FMOs, CYP450 also needs NAD(P)H as a hydrogen donor, and oxygen molecules to catalyze the oxidation of the substrates. Besides, in the CYP450 catalytic cycle, NAD(P)H also serves as the direct electron donor to the enzyme. The molecular weights of the two enzymes are alike, and the chemical structures of the substrates they catalyze are also very similar. The main differences between the two families of enzymes include catalytic activity, stability of the intermediates, and the mechanism of the catalytic cycle. The C4a hydroxy-peroxide used by FMOs to catalyze the substrate is a very stable intermediate, which can stay for several hours at 4 °C in the cavity of FMO, and scientists believe that this intermediate exists in most organisms containing FMO [32]. In terms of catalytic activity, they are also very different. Even if the same substrate is consumed to react with the two enzymes, the products are usually distinctive. Compared with CYP450 enzymes, FMOs produce less toxic substances with higher catalytic efficiency [33]. During the catalytic cycle, FMOs do not need additional redox partners to participate in the cycle or activate the substrate [31]. In addition, FMOs can be heterologously expressed in prokaryotic hosts. Almost every CYP450 enzyme has a specific mechanism-based inhibitor, but FMOs have no such type of inhibitors. These advantages also greatly expand the application range of FMOs [11,16].

**Figure 1:**
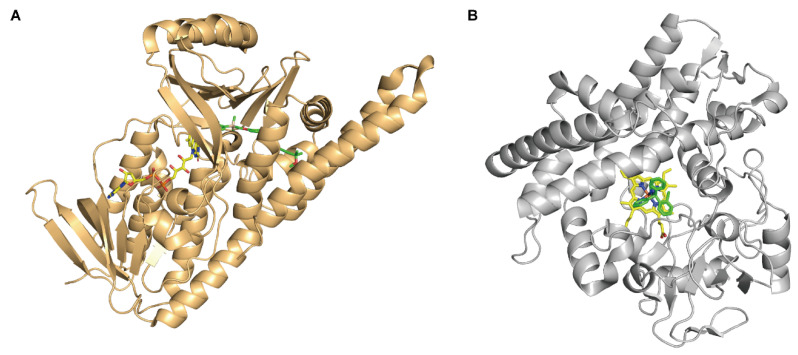
Crystal structures of human SQLE (**A**) and OleP from *Streptomyces antibioticus* (**B**). SQLE is presented as cartoons in orange. FAD (yellow) and NB-598 (green) are shown as sticks. OleP is presented as cartoons in grey. Heme (yellow) and CTZ (green) are shown as sticks.

In order to reveal the detailed difference between FMOs and CYP450, the crystal structures of human squalene epoxidase (SQLE), and OleP, a cytochrome P450 epoxidase from *Streptomyces antibioticus,* are compared. The overall folding of the two structures is different. For SQLE, its FAD-binding domain adopts the GR2 Rossman fold, while its substrate-binding domain adopts a two-layer βα sandwich domain with seven-stranded β-sheet structure, followed by two helices at the C-terminus [34]. For OleP, its secondary structural elements consist of 15 helices and 2 β-sheets spatially arranged in the typical prism-like fold of cytochrome P450s [35]. The cofactor bound to SQLE is FAD, while it is heme for OleP, determining the different catalytic mechanisms for the two epoxidases. The substrate binding domain is fused to the FAD binding domain in SQLE. However, there is no separate domain for the heme and the substrate (Figure 1).

## 4. The Substrate Specificity of FMOs

Ziegler et al. found that FMOs were promiscuous for substrates when studying the purified porcine liver FMO (FMO1). Generally, compounds containing soft nucleophiles that can approach the flavin cofactor in FMO can be used as substrates, such as nitrogen-containing and sulfur-containing compounds. At the same time, they found that positively and negatively charged compounds have a decisive impact on the reactivity of the substrates. For example, compounds containing a single positive charge are usually excellent substrates that can bind well to FMO and be oxidized. However, compounds with negative charges react with FMO or have very poor reactivity [36,37,38], but there are exceptions, such as sulfur and lipoic acid. It must be emphasized that the positive charge is limited to a single charge, and compounds with more than one positive charge cannot usually react with FMOs [39,40,41].

However, caution must be exercised in inferring the substrate specificity of a particular FMO across species, because even when the same substrate is consumed to interact with FMOs from different sources, the reactivity may be different, or even show major differences. For example, Tynes et al. found that both chlorpromazine and imipramine could be oxidized by pig liver FMO, but not rabbit lung FMO [42]. Then they used a variety of substrates including sulfur to test the substrate specificity of the two and found that there were great differences in substrate specificity [43]. In order to explore the underlying causes of the substrate specificity of FMOs from different species, Nagata and other researchers proposed in 1990 that the size of the substrate inlet channel of rabbit lung enzyme and pig liver enzyme may be the key factor influencing their FMO activity. They believed that it was precisely because of the size difference of the substrate inlet channel that the substrate is difficult to approach C4a-(hydrogen) peroxide [43,44,45]. In addition to the charge and substrate inlet channels mentioned above, researchers believe that there are other factors affecting the substrate specificity of FMO enzymes [46,47,48,49,50]. Limited by current technical methods, these factors cannot be determined at present, but it is believed that, with the continuous development of molecular and structural biology, the mystery of substrate specificity hidden behind FMOs will be eventually uncovered [51].

## 5. Reactions Catalyzed by FMOs in Natural Product Biosynthesis

NPs play an important role in drug discovery because of their unique structures compared with those of traditional drugs [52,53,54]. Drugs derived from natural products have more diverse scaffolds and functional groups which endow them with a variety of biological activities [55]. FMOs are a widely distributed enzyme family that can catalyze the redox reaction of a variety of substrates [56]. FMOs share special protein structures and unique mechanisms for catalyzing substrate oxidation. FMOs stabilize the internal C4a-hydroperoxide flavin intermediate at special sites, and they catalyze the synthesis of a variety of substances including natural products through a unique catalytic mechanism. The reactions involved in the synthesis of natural products catalyzed by FMOs include hydroxylation, epoxidation, Baeyer-Villiger oxidation, oxidative decarboxylation, halogenation, and sulfur oxidation [57,58].

Meanwhile, the continuous development of protein engineering and evolutionary strategies promotes the manipulation of enzymes in heterologous expression systems to produce various natural products with complex structures [59]. These chemical scaffolds are difficult to obtain by chemical methods. Compared with organic synthesis methods, enzyme catalyzed synthesis of natural products has some unparalleled advantages [60], such as safety, sustainability, simple procedure, and high enantioselectivity. With the direction of the gradually deepening research into FMOs, it is approaching industrial application to make use of the special catalytic potential of FMOs to produce intermediates with special structures, especially some enantioselective compounds that are difficult to obtain by chemical methods [58].

### 5.1. The Biosynthesis of Natural Products through Dearomatization Catalyzed by FMOs

A benzene ring is a six membered carbon atom ring with an alternating single bond and double bond, which displays high resonance energy. Therefore, the benzene ring is very stable, and it is generally difficult to dearomatize and destroy in aromatics. However, in the synthesis of natural products with bioactivity, it is a very useful strategy to construct special chemical frameworks by de-aromatization of aromatics. In the field of organic synthesis, transition metals generally catalyze the de-aromatization of aromatic compounds, but these transition metal catalysts usually have many disadvantages. For example, in the process of catalytic aromatization, a certain amount of catalysis is usually required to achieve the conversion of substrates, and some byproducts will be produced with the aromatization of substrates [61]. In addition, it is difficult to achieve accurate site selectivity and stereoselective oxidative for de-aromatization control by transition metal catalysts. However, it is promising that scientists have found that there are many microorganisms involved in de-aromatization in nature. These microorganisms usually carry out redox reaction on aromatic and heteroaromatic derivatives through oxygenase or reductase, so as to realize substrate de-aromatization [62].

FMOs are involved in the oxidative de-aromatization of many substances in nature [63]. Compared with traditional transition metal catalysts, the FMO oxidative de-aromatization platform has unparalleled advantages, including mild reaction conditions, accurate site selectivity and stereoselectivity, as well as avoiding environmental pollution caused by toxic transition metal catalysts [63,64].

**Scheme 5:**
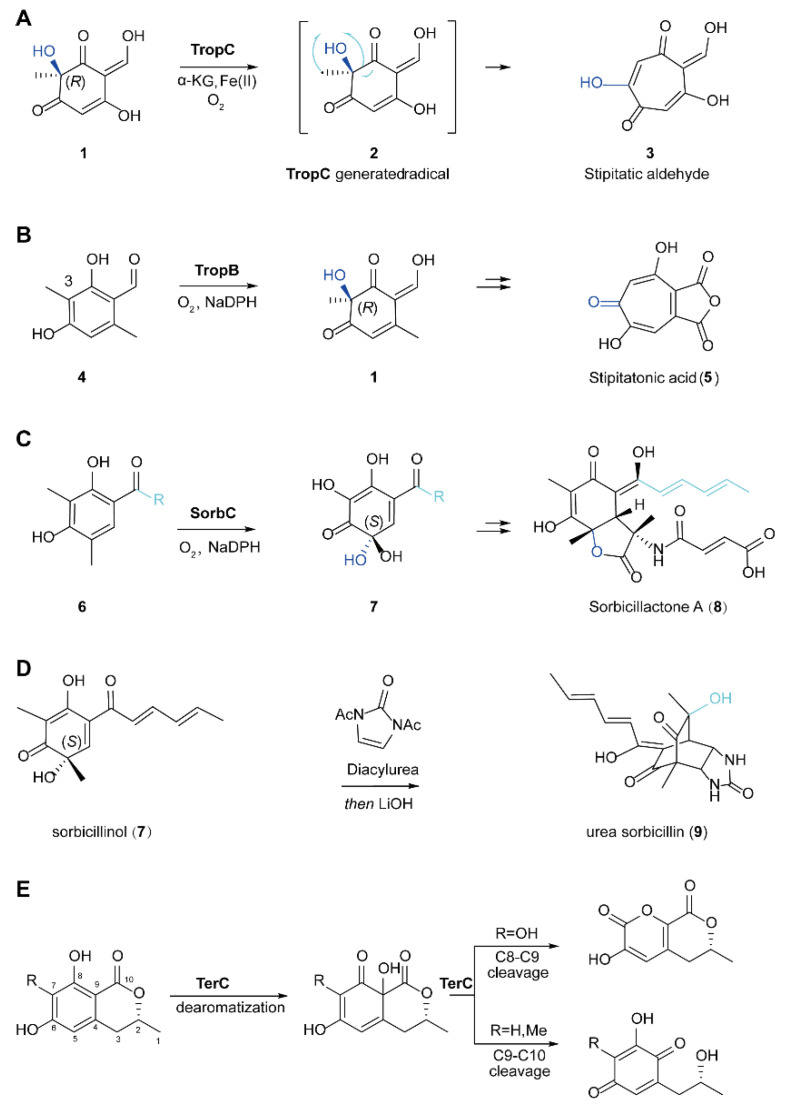
(**A**) FMO TropC is involved in the enzymatic total synthesis of stipitatic aldehyde. (**B**) FMO TropB is involved in the enzymatic total synthesis of Stipitatic acid. (**C**) FMO SorbC is involved in the enzymatic total synthesis of Sorbicillactone A. (**D**) Chemoenzymatic synthesis of urea sorbicillinoid. (**E**) TerC catalyzed oxidative de aromatization reactions of 6-hydroxymellein (6-HM) analogues.

Tropolones are a type of natural product from bioactive fungi with diverse structures. They were isolated from *Penicillium* by Raistrick et al. in 1942. However, limited by the detection methods at that time, the exact structural formula of this substance was not known. It was not until 1945 that Dewar boldly proposed the unprecedented structure of this substance characterized by an aromatic cycloheptatriene core bearing an α-hydroxyketone functionality. Dewar later named these seven-membered ring compounds tropolones. The discovery of tropolones paved a new way for scientists at that time to understand aromaticity and bonding [65]. The method of organic synthesis of tropolones mainly depends on the ring expansion reaction [66] by double electron rearrangement or the free radical based method [67] from ortho-dearomatized catechols. The biosynthesis pathway of tropolones was cleverly designed by organisms through FMOs [68,69]. Narayan et al. skillfully synthesized the natural product of hydroquinone stipitatic aldehyde through a two-step biocatalytic cascade reaction by using resorcinol derived quinone 1 as substrate and FMO 3-methylorcinaldehyde monooxygenase (TropB) as catalyst (Scheme 5) [70]. First, **4** was oxidatively dearomatized by TropB. After de-aromatization, quinone intermediate **1** was obtained. Then **1** underwent a second oxidation catalyzed by non heme iron monooxygenase 2-oxoglutarate-dependent dioxygenase (TropC). The researchers speculated that the mechanism of this process might be the rearrangement reaction containing one electron, followed by the rebound and elimination of oxygen, and finally the double electron rearrangement reaction and the elimination reaction participated by iron, leading to the net ring expansion and finally the formation of Stipitatic aldehyde **3** [71].

In 2021, Shu et al. reported that flavin monooxygenase TerC (encoded by the *terCDEF* gene) catalyzed a series of oxidative de aromatization reactions of 6-hydroxymellein (6-HM) analogues. After the de-aromatization reaction, flavin further induces different skeleton deformation of the de-aromatization reaction products through bimodal reaction cascades to form benzoquinone or pyrone, which is only regulated by the substituent at C7 position. These studies revealed the strong catalytic ability of FPMO TerC and provided a powerful biocatalyst for the degradation of waste aromatic pollutants in nature [72].

In addition, FMO is also involved in the biosynthesis of natural products of sorbicillinoid urea. Sorbicillin urea **9** was isolated from the fungus *Paecilomyces marquandii* by Cabrera et al. It was found that this chemical has good antibiotic activity [73]. In 2017, Narayan et al. used FMO dihydro-sorbicillin monooxygenase (SorbC) to catalyze the oxidative de-aromatization of resorcinol substrate **6** (Scheme 5). After oxidation, **6** was converted to sorbitol **7**. Then, bi-sacylated urea was supplemented to the system, resulting in the [4+2] cycloaddition reaction. After this reaction was completed, LiOH was finally added to the reaction system, and finally the target product urea sorbitol natural product **9** was obtained, and the total yield of the three steps was 21% [69].

### 5.2. The Biosynthesis of Cytochalasin Natural Products Catalyzed by FMOs

Cytochalasin was first isolated by Rothweiler and Tamm in 1966. So far, a large number of this family of natural products have been identified [74]. Cytochalasin is produced by a variety of polyketide synthase and non-ribosomal peptide synthases (PKS-NRPs). It has a wide range of unique activities, including antibacterial, antiparasitic, and anticancer activities, and some compounds of this family are considered to be phytotoxins [75,76]. The most essential characteristic of its structure is that it has a tricyclic core, and the macrocycle is fused with a bicyclic lactam (iso-indolone). The major species of cytochalasin producing bacteria include 10 species [74], such as *Penicillium*, *Aspergillus,* and *Zygosporum*. Cytochalasin (**10**) is produced by *Aspergillus clavatus.* Studies have shown that cytochalasin E (**10**) is a potential angiogenesis inhibitor. Cytochalasin E (**10**) contains a vinyl carbonate part. However, the non-enzymatic conversion of ester to carbonate is a very challenging synthetic transformation.

**Scheme 6:**
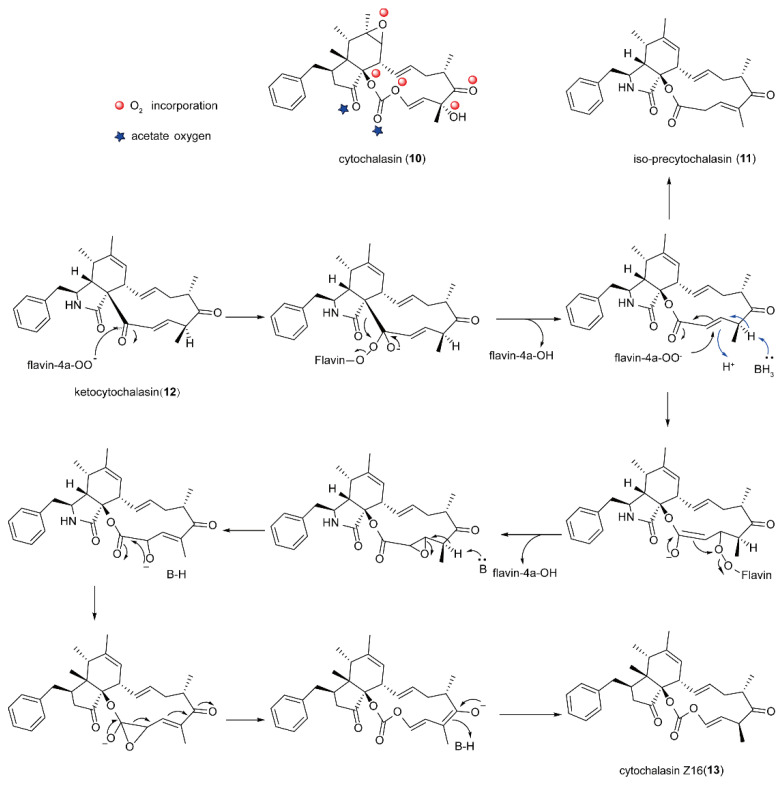
FMO CcsB catalyzes the conversion of macro-ketone intermediate keto-cytochalasin **12** to lactone **11** and the corresponding carbonate **13** during the enzyme-promoted formation of cytochalasin **10**.

Tamm et al. summarized previous relevant studies of cytochalasin in the mid-1970s. For example, microorganisms can produce testosterone lactone from androsterol+ene-3,17-dione by enzymatic Baeyer-Villiger oxidation, as well as testosterone acetate from progesterone by the same methods [77]. On this basis, they proposed that macrocyclic ketone precursors produce cytochalasin through enzymatic Baeyer-Villiger oxidation to produce macrolides [78]. FMO is a common enzyme for Baeyer-Villiger oxidation in vivo [19,79,80]. In 2014, Liu et al. studied the cytochalasin E biosynthesis gene and showed that cytochalasin Baeyer-Villiger monooxygenase (CcsB) is an FMO, which can play the function of a Baeyer-Villiger monooxygenase’s (BVMO’s) catalyst, oxidize macrocyclic precursors, and introduce a carbonate structure. They also predicted that, in the synthesis of all cytochalasin containing an ester group, there must be a CcsB-like enzyme to perform the first Baeyer-Villiger oxidation and introduce the carbonate part into the structure. Liu et al. found that the partially oxidized macrocyclic ketone **12** was transformed into the corresponding lactone **11,** according to the classical BVMO mechanism under the action of FMO CcsB (Scheme 6), and the group migration followed the rules of migration ability, and then transformed into allyl carbonate **13**. Because single-bond carbonate oxygen comes from different O_2_ molecules, CcsB must reload oxygen before the second oxidation. They believe that, through the Michael addition reaction with the participation of peroxy-flavin anion (Fl-4a-OO^−^), the second oxygen can be inserted into the β-carbon of vinyl ester, in which the vinyl ester part of the double bond is then converted into α-β epoxide, and then undergoes a ring opening rearrangement and other processes to afford the final target product [81].

Most notably, in 2021, Ebrahimi et al. conducted computational analysis on the inhibitory effect of fungal secondary metabolites on the RNA dependent RNA polymerase of SARS COV-II, because coronavirus disease (COVID-19) was spreading rapidly all over the world and endangering the lives of tens of thousands of people. In this study, they mainly used docking and molecular dynamics simulation methods. The inhibitory potential of various secondary metabolites to New Coronavirus RNA dependent RNA polymerase (RdRp) was studied, and the selected compounds were further evaluated by molecular dynamics (MD) simulation. It was found that 18 methoxy-cytochalasin J (MCJ) and pyrrolidine A are more effective virus inhibitors than other compounds, so they can be used for further experimental research [82].

### 5.3. The Synthesis of Natural Products of Chaetoglobosin A Catalyzed by FMOs

NPs of fungi usually present important medicinal biological activities, for example, Chaetoglobosin A, a cytotoxic metabolite of *Chlorella vulgaris* [80,83,84]. Chaetoglobosin compounds have a wide range of related biological activities against the growth of cyto-skeletals, and also have a certain cytotoxicity to plants, as well as antibiotic activities. Chaetoglobosin A can also cause multinucleation and multipolar division of HeLa cells, and studies have shown that it can cause acute toxicity in mice [85].

Watanabe et al. predicted and identified the gene cluster responsible for the biosynthesis of Chaetoglobosin A in *Penicillium expansum*. They found that three overlapping cosmoid genes can construct the core structure through the interaction of the encoded rare fungal PKS-NRPs hybrid synthase, PKS-NRPs, and an independent enol reductase (CheB). In order to understand the synthetic pathway of the natural products of the Chaetoglobosin family, they established a strain of *Clostridium globosum* with a DNA ligase ligD deficiency, since, for a long time scientists had found that *Clostridium globosum* will produce Chaetoglobosin natural products, Therefore, they carried out targeted gene disruption using DNA ligase ligD deficiency *Clostridium globosum* strains [86,87,88]. Each gene in the Chaetoglobosin A biosynthesis gene cluster was knocked out by targeted homologous recombination. Finally, the genes encoding oxidoreductases were determined, which were an FMO (CHGG_01242-2) and two CYP450 oxygenases [89]. At present, about 40 types of Chaetoglobosin and their analogues have been isolated and identified [90].

**Scheme 7:**
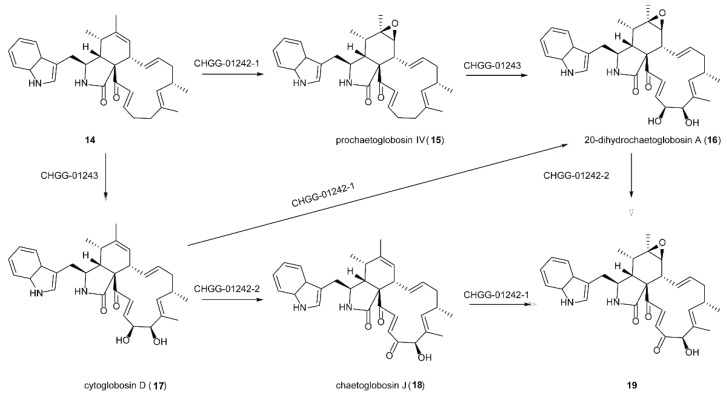
Biosynthetic Pathway for the Transformation of **14** to **19** Involving Multiple Oxidation Steps.

Their studies showed that FMO plays an important role in catalyzing the core structural formation of the natural products of Chaetoglobosin. For example, during the synthesis of **19** (Scheme 7), FMO (CHGG-01242-1) can selectively oxidize one of the orthodi-hydroxy groups in the substrate to produce the structure of o-carbonyl ketone. Meanwhile, Watanabe et al. also studied the synthetic pathways of other bioactive compounds with important values, and FMO is involved in the synthetic pathways of these active compounds, such as pseurotin, a secondary metabolite of fungi [91,92,93,94].

### 5.4. Biosynthesis of Polyether Natural Products Catalyzed by FMOs

**Scheme 8:**
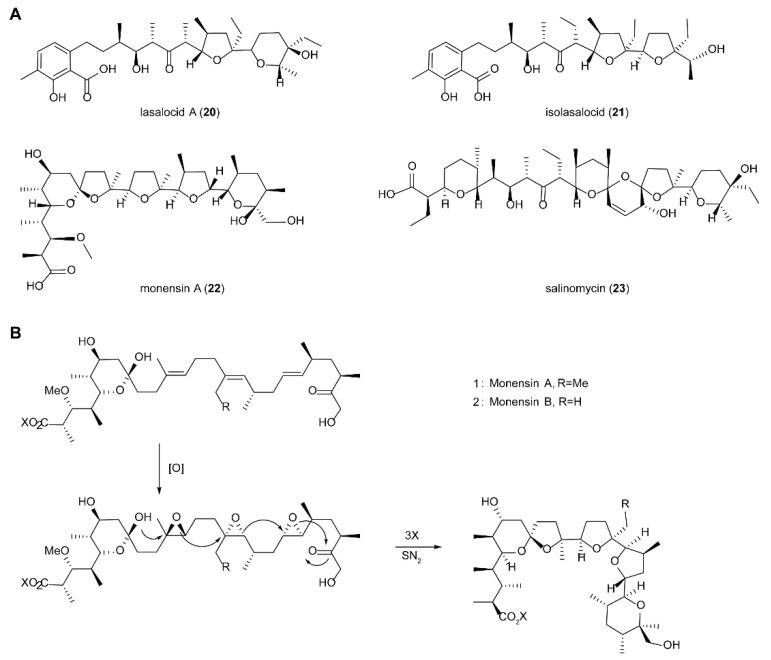
(**A**) Chemical structures of some representative polycyclic polyether natural products. (**B**) CCW model.

Natural polycyclic poly-ethers, such as ionophore poly-ethers, antitumor acetyls, polyether ladders, and poly-epoxy-quinoline are compounds with branched poly-oxy-carboxylic acid structures (Scheme 8A). The polycyclic polyether scaffold is formed by the catalysis of a multifunctional enzyme, modular type I polyketide synthase (PKS) [95,96]. The building blocks of a natural polycyclic polyether skeleton are acetate, propionate, and butyrate [97]. Polycyclic poly-ether structures usually contain multiple asymmetric centers, which are generally rich in oxygen atoms, and there are multiple tetrahydrofuran and tetrahydropyran rings. This unique structure makes it easy to transport metal ions. Metal ions are generally chelated by oxygen atoms in poly-ethers and their outer surface is hydrophobic. Therefore, their biological activity mainly affects the permeability of ion transport in biofilm, thus having good antibiotic activities [98].

For example, Monensin and Lasalocid A [99] are ion carrier poly-ethers and are the main drugs for controlling poultry and poultry coccidiosis, which can improve feed efficiency for ruminants. They can also be applied as anticancer, antimalarial, and anti-human immunodeficiency virus agents [100,101,102]. In 1983, Cane, Celmer and Westley proposed a simple but universal natural polycyclic polyether biosynthesis model (CCW model) (Scheme 8B) [97]. This hypothesis shows that achiral polyenes undergo a continuous enantioselective reaction, i.e., epoxidation, to install all necessary stereochemistry, and then the resulting poly-epoxy compound is used as the substrate of a ring opening cascade to obtain the target polyether [103].

**Scheme 9:**
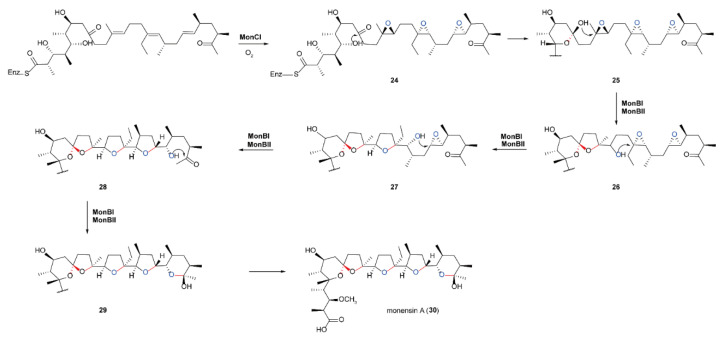
Mechanism of polyether formation during Monensin biosynthesis involving MonBI/MonBII.

In 2001, Leadley et al. discovered the Monensin biosynthesis gene cluster [98]. Monensin is produced by actinomycete *Streptomyces Cinnamomum*, which can selectively bind sodium ions. They found an FMO MonCI and a pair of epoxide hydrolases MonBI and MonBII genes. Gene inactivation studies showed that the FMO MonCI participates in the stereoselective epoxidation of three double bonds in the substrate, forming three ethylene oxide structures. Then a pair of MonBI/MonBII catalyzes the ring opening cascade reaction of epoxides to obtain Monensin [104,105]. This study strongly proves the CCW model (Scheme 9).

**Scheme 10:**
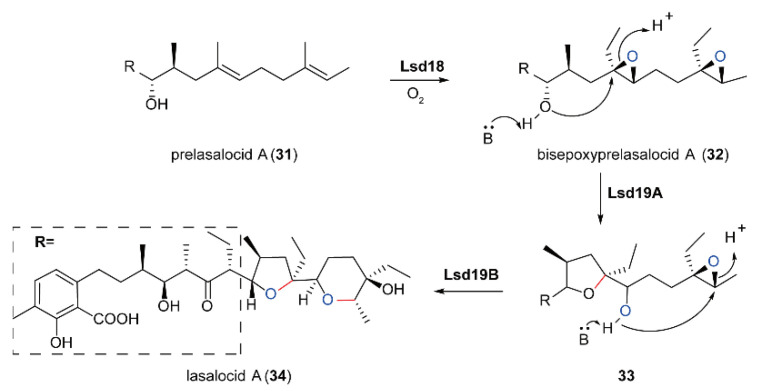
FMO **Lsd18** catalyzes the formation of epoxide intermediates to obtain cyclic poly-ether Lasalocid A.

**Scheme 11:**
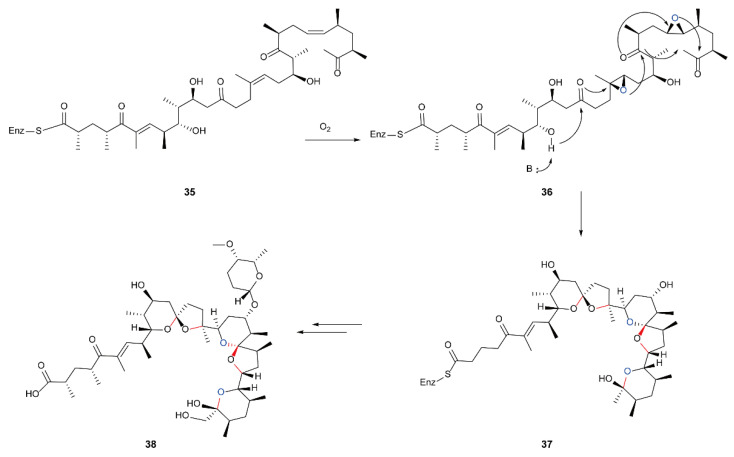
Mechanism of Polyether Formation in Nanchangmycin Biosynthesis.

Lasalocid A (**34**) is the simplest polycyclic polyether ionophore, which contains a THF and a THP ring [106]. Michael et al. proposed the detailed biosynthesis process of Lasalocid as shown (Scheme 10). Similar to Monensin biosynthesis, Lasalocid A also contains a flavin dependent cyclooxygenase Lsd18 and an epoxide hydrolase Lsd19. Flavin dependent cyclooxygenase Lsd18 first epoxidates the two E-double bonds in pre-lasalocid A in a stereoselective manner, and the epoxidation is carried out from the terminal double bond to the inner double bond to afford the epoxide. Then, epoxide hydrolase Lsd19 catalyzes two rounds of epoxide ring opening reactions of two epoxides in a highly regioselective manner [107]. Nanchangmycin (**36**) (Scheme 11) also belongs to the natural polycyclic polyether family, and its enzymatic synthesis strategy is similar to the above mechanism. FMO not only catalyzes epoxidation to form polycyclic polyether natural products but also catalyzes the formation of other polycyclic natural products.

**Scheme 12:**
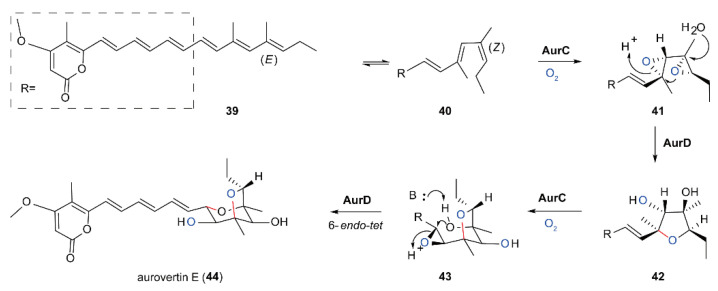
FMO AurC is involved in the enzymatic total synthesis of Aurovertin.

Aurovertin is an antibiotic from the fungus Calcarisporium arbuscula. Its main function is to inhibit oxidative phosphorylation, thus having strong cytotoxicity [108,109]. It was found that FMO Aurora kinases (AurC) is required in the biosynthesis of aurovertin [110]. This enzyme participates in the three-step epoxidation near the terminal double bond in the substrate **39** (Scheme 12). In the first step of the reaction, it catalyzes the stereoselective epoxidation of the first two double bonds of (E,Z) terminal diene **40** to produce bi-sepoxide **41**; then, epoxide hydrolase Aurora kinases (AurD) catalyzes the epoxide ring opening cascade reaction of bi-sepoxide **41** to produce di-hydroxyfuran **42**. Subsequently, the double bond close to THF ring in di-hydroxyfuran **42** is converted to **43** by FMO AurC. The intramolecular anti Baldwin 6-endo-tet cyclization of **43** catalyzed by FMO AurD generates the octane ring support of 2,6-dioxa bi-cyclo [3.2.1] **44**.

**Scheme 13:**
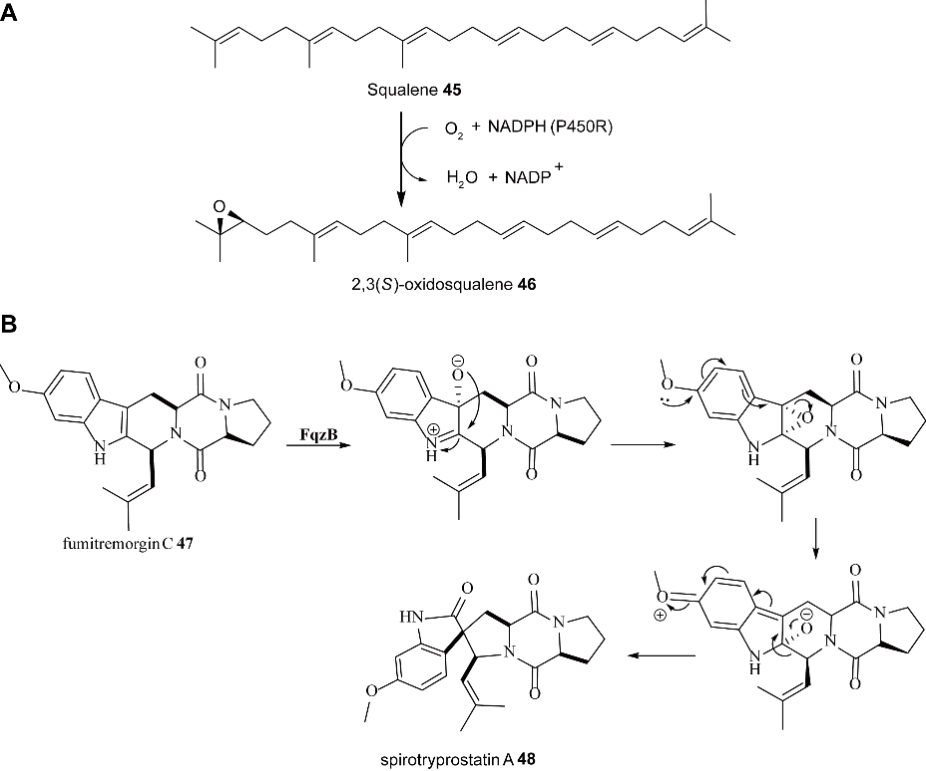
(**A**) Squalene mono-oxygen as catalyzed the formation of 2,3(*S*)-oxidative squalene **46**. (**B**) Proposed reaction mechanism for the catalytic spiro-plast prostaglandin A **48** formation of FqzB.

In addition, Padyana et al. reported their studies on human squalene monooxygenase in 2019, in which human squalene monooxygenase can catalyze the stereo conversion of squalene **45** to 2,3 (S)-oxidized squalene **46** (Scheme 13A), which is a key step in cholesterol biosynthesis [34]. In 2020, Matsushita et al. studied the structure and function of epoxidase FqzB (encoded by the *fqzB* gene), which catalyzes the spiro carbon formation in the biosynthesis of natural products of fungi. They also characterized the kinetics of FqzB and its site-specific mutants. Fqzb is an FMO from the biosynthesis pathway of fumizoline. It can epoxidize fumizoline F and form spiroplast prostaglandin A by epoxidizing the precursor fumizolin C (Scheme 13A) [111].

### 5.5. The Synthesis of Chiral Sulfoxide Compounds Catalyzed by FMOs

It is well known that stereoselective oxidation of substrates by biocatalysts has a very broad application potential in industry [112,113]. Baeyer-Villiger monooxygenases, a family of enzymes with wide application, can catalyze the chemo-, regio- and enantio-selective oxidation of substrates. In addition to catalyzing the biological conversion of aldehydes and cyclones to corresponding esters and lactones, it can also improve the ability of epoxidation of heteroatoms (sulfur, nitrogen and boron) to construct the core skeleton in the synthesis of many complex natural products [114,115,116,117,118]. However, at the same time, BVMOs also have many problems in practice. For example, they usually need stoichiometric O_2_ and NAD(P)H in the process of catalysis. The reduced nicotinamide coenzyme NAD(P)H is expensive [119], and this disadvantage greatly limits its application in industrial catalysis. In order to overcome this problem, several electrochemical and photochemical methods have been explored, but with low efficiency [120,121,122].

In addition, whole cells were used to try to regenerate the coenzyme [117]. The advantage of this method is that it can utilize the host to regenerate the coenzyme, avoid the laborious purification step of FMO, and make use of the host’s coenzyme regeneration ability. The results showed that the whole cells were an effective catalyst for Baeyer-Villiger oxidation [123,124,125,126,127], but this method also has limitations, including cytotoxicity, inhibition of substrate/product binding to the enzyme, and poor degradation and oxygen transfer rate of the product [128]. In 2011, Fraaije et al. proposed covalent fusion of FMO BVMOs and soluble NADPH regenerated phosphite dehydrogenase (PTDH) from *Pseudomonas stutzeri* to prepare self-sufficient monooxygenases, including a bifunctional biocatalyst PTDH-mFMO. Among them, mFMO was proved to be able to transform endogenous indole in *E. coli* cells [116]. The advantages of these bifunctional biocatalysts are that less enzyme is required in the whole catalytic process with fewer enzyme separation procedures. In addition, this bifunctional catalyst greatly promotes the regeneration and utilization of coenzymes. Therefore, it can be applied to the conversion process of a variety of substrates [129]. The experimental results showed that most aromatic sulfides could be oxidized to sulfoxide with good catalytic efficiency.

**Scheme 14:**
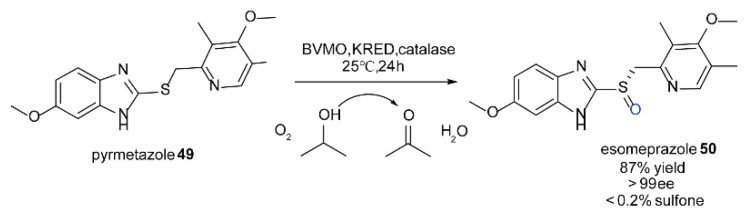
BVMO-FAD catalyzes the conversion of pyrmet-azole **49** to esomeprazole **50**.

Cyclohexanone monooxygenase (CHMO) was first found in *Acinetobacter sp* [130]. This is a flavin monooxygenase because it can catalyze the Baeyer Villiger oxidation of cyclohexanone to obtain cyclo-prolactone [117]. As a result, it is also called Baeyer Villiger monooxygenase (BVMO). In addition to catalyzing a variety of oxidation reactions, BVMO can also be modified to catalyze the oxidation of sulfur to sulfoxide [131]. In 2018, Bong et al. carried out directed evolution of BVMO and selected ketone reductase (KRED)-mediated oxidation of propyl-2-alcohol (IPA) to effectively realize the regeneration of NADPH cofactors, so that it can efficiently catalyze the oxidation of pimetazole to esomeprazole with higher catalytic efficiency and higher chemical and enantioselectivity (Scheme 14) [132].

In 2019 [133] and 2020 [134], Zhang et al. successively modified the cyclohexanone monooxygenase (AcCHMO) in *Acinetobacter calcoaceticus* by structure-based protein engineering method to a pyrmet-azole monooxygenase that can catalyze the oxidation of pyrmet-azole to esomeprazole, and named it AcPSMO. Some limiting factors encountered in the scale-up of the modified reaction were studied, and then the biological oxidation reaction was scaled up in a step-by-step manner for the preparation of esomeprazole [15].

### 5.6. The Biosynthesis of Auxin Natural Products Catalyzed by FMOs

Trp dependent auxin is an important plant hormone, which has a vital impact on many processes of plant growth and development such as cell division and elongation, differentiation, embryogenesis, seedling growth, flower development, abscission, and flowering [135,136,137]. Indole-3-acetic acid (IAA) is considered to be the most important auxin in plants. It has several synthetic pathways. For example, there are four pathways for Trp dependent biosynthesis of IAA in plants: (I) the YUCCA(YUC) pathway, (II) the indole-3-pyruvate (IPA) pathway, (III) the indole-3-acetamide (IAM) pathway, and (IV) the indole-3-acetaldehyde oxime (IAOx) pathway [138].

**Scheme 15:**
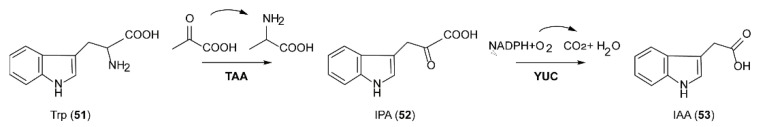
The Tryptophan-Dependent Auxin Biosynthesis Pathway. The first step is catalyzed by **TAAs** that transfer the amino group from Trp to an alpha keto acid such as pyruvate to generate IPA and another amino acid. The second step is an oxygen and NADPH-dependent reaction catalyzed by the **YUC** flavin-containing monooxygenases.

The YUC pathway is considered to be a common IAA biosynthesis pathway, which exists in many plants. YUC is an FMO, and its gene is mainly distributed in the meristem, juvenile primordium, vascular tissue, and reproductive organs. It was identified as a key auxin biosynthetic enzyme for the first time in 2001 [138], as feeding and biochemical studies had shown that overexpression of the YUC gene led to excessive production of auxin. FMO YUC catalyzes the hydroxylation of the tryptamine amino group, which is also the rate limiting step of tryptophan dependent auxin biosynthesis. At the same time, genetic analysis has clearly shown that Arabidopsis tryptophan aminotransferase (TAA) and YUC flavin monooxygenase-like proteins are two families necessary for IAA biosynthesis in plants. Transaminase TAA can catalyze L-tryptophan (L-Trp) to produce indole-3-pyruvate (IPA) which is subsequently catalyzed by the FMO of the YUC family to produce IAA (Scheme 15). The expression patterns of TAA and the YUC family are spatially regulated in plant development. The two proteins usually work together to regulate the production of IAA, which plays an important role in many developmental processes [138,139].

In 2020, Uc-Chuc et al. found that Yucca mediated auxin IAA biosynthesis also plays an essential role in the induction of the coffee canephora cell embryo. They observed that IAA belongs to the de novo biosynthesis during cell embryo development. Later, they demonstrated by QRT PCR that the expression level of Yuc gene was consistent with the free IAA signal found in explants during cell embryo induction [140].

## 6. Outlook

Natural products have played more and more important functions in drug discovery. It is well known that natural products are the major resource for anti-infection medicines such as antibiotics. Currently, as COVID-19 is threatening human lives, it is imminent to seek new medicine to fight against this virus. Apart from this, other diseases such as cancer also require more efficient medications. With this background, it will be very important to develop a biological enzymatic natural product synthesis platform to obtain complex natural products with various unique scaffolds in order to greatly accelerate the development and discovery of drugs based on natural products. In the process of natural product biosynthesis, oxidoreductase plays an important role in structural complexity among which FMO introduces complex structures into the substrate by generating reactive (hydrogen) peroxide flavin, so as to endow natural products with diverse biological activities. FMO is an excellent multifunctional oxidoreductase, which can catalyze the formation of different types of C-O bonds [141,142], including hydroxylation, Baeyer-Villiger oxidation, sulfur oxidation, epoxidation, and other reactions with high chemo-, regio- and stereo-selectivity. Although the number of FMOs in catalyzing the synthesis of natural products is relatively small compared with the CYP450 family [143,144], FMO has unique advantages such as producing fewer toxic substances and being self-sufficient in the process of catalysis with higher efficiency [145].

Although significant progress has been made in understanding the catalytic mechanism and reaction types of FMOs, there are still many problems unsolved, which must be the focus of future research. For example, how is the oxygen transferred to the xanthin cofactor, and does FMO control the regioselectivity of N5 and C4a by strictly directing O_2_ to reach proximity to the xanthin cofactor? Are there any other influencing factors [145]? However, with the rapid development of biochemistry and structural biology, as well as the progress of synthetic control of gene expression and effective genome editing methods, more detailed catalytic process of FMOs will be revealed to further promote the application and development of FMO enzymes for the production of novel medicines [146].
